# Are Behavioral Ecotoxicity Endpoints Relevant at the Population Level? Evidence-Based Insights for Environmental Protection

**DOI:** 10.1021/acs.est.5c07777

**Published:** 2025-12-24

**Authors:** Michael G. Bertram, Marlene Ågerstrand, Sigal Balshine, Jack A. Brand, Bryan W. Brooks, ZhiChao Dang, Alex T. Ford, Henner Hollert, Matthew K. LeFauve, Jack L. Manera, Jake M. Martin, Marcus Michelangeli, Maria Moiron, Eleanor R. Moore, Holly J. Puglis, Andrew Sih, Jeffery A. Steevens, Eli S. J. Thoré, Bob B. M. Wong, Lauren Zink, Tomas Brodin

**Affiliations:** † Department of Wildlife, Fish, and Environmental Studies, 8095 Swedish University of Agricultural Sciences, Skogsmarksgränd 17, Umeå 907 36, Sweden; ‡ Department of Zoology, Stockholm University, Svante Arrhenius väg 18b, Stockholm 114 18, Sweden; § School of Biological Sciences, 2541 Monash University, 25 Rainforest Walk, Melbourne 3800, Australia; ∥ Department of Environmental Science, Stockholm University, Svante Arrhenius väg 8c, Stockholm 114 18, Sweden; ⊥ Department of Psychology, Neuroscience, & Behaviour, McMaster University, 1280 Main Street West, Hamilton, Ontario L8S 4K1, Canada; # Institute of Zoology, Zoological Society of London, Outer Circle, Regent’s Park, London NW1 4RY, U.K.; ∇ Department of Environmental Science, 14643 Baylor University, One Bear Place, Waco, Texas 76798-7266, United States; ○ National Institute for Public Health and the Environment (RIVM), Antonie van Leeuwenhoeklaan 9, Bilthoven 3721 MA, The Netherlands; ◆ Institute of Marine Sciences, School of Biological Sciences, 6697 University of Portsmouth, Ferry Road, Portsmouth PO4 9LY, U.K.; ¶ 9173 Goethe University Frankfurt, Max-von-Laue-Straße 13, Frankfurt am Main 60438, Germany; †† Superfund & Emergency Management Division, U.S. Environmental Protection Agency (EPA), 5 Post Office Square, Boston, Massachusetts 02109, United States; ‡‡ School of Life and Environmental Sciences, Deakin University, 75 Pigdons Road, Waurn Ponds 3216, Australia; §§ Australian Rivers Institute, Griffith University, 170 Kessels Road, Nathan 4111, Australia; ∥∥ Department of Evolutionary Biology, 9167 Bielefeld University, Konsequenz 45, 33615 Bielefeld, Germany; ⊥⊥ Joint Institute for Individualisation in a Changing Environment (JICE), Bielefeld University and University of Münster, Konsequenz 45, 33615 Bielefeld, Germany; ## 2928 U.S. Geological Survey (USGS), Columbia Environmental Research Center, 4200 New Haven Road, Columbia, Missouri 65201, United States; ∇∇ Department of Environmental Science and Policy, University of California, 350 E Quad, Davis, California 95616, United States; ○○ Laboratory of Adaptive Biodynamics, Research Unit of Environmental and Evolutionary Biology, Institute of Life, Earth, and Environment, University of Namur, Rue de Bruxelles 61, Namur 5000, Belgium; ◆◆ TRANSfarm, Science, Engineering, and Technology Group, KU Leuven, Bijzondereweg 12, Bierbeek 3360, Belgium; ¶¶ Department of Zoology, University of British Columbia, #3051−6270 University Blvd., Vancouver, British Columbia V6T 1Z4, Canada

**Keywords:** behavior, chemical, ecology, ecotoxicology, hazard assessment, regulation, risk assessment

## Abstract

A substantial body of evidence exists demonstrating that exposure to environmental contaminants can alter animal behavior. Moreover, methodological and technological advancements, as well as increasing standardization, mean that behavioral ecotoxicity studies are more rigorous and reliable than ever before. Despite this, behavioral data are still seldom used in the risk assessment and regulation of chemicals. This is partly due to a lack of clarity among some stakeholders about whether changes in behavior at the individual level result in population-level outcomes. To address this, we first consider the state of evidence within the field of behavioral ecotoxicology linking individual-level behavioral alterations with population-level consequences. We then assess the evidence from behavioral ecology and other neighboring fields that supports this link. Further, we evaluate whether some behavioral endpoints are more easily tied to population-level changes than others. In this regard, we propose combining insights from two complementary ecological frameworks—the functional trait framework and the limiting traits framework—to evaluate which behaviors should be prioritized in ecotoxicological research and regulatory efforts. We contend that the link between behavioral changes and population-level outcomes is evident, with behavioral endpoints representing a highly valuable yet so far underutilized line of evidence in applied environmental protection.

## Graphical abstract

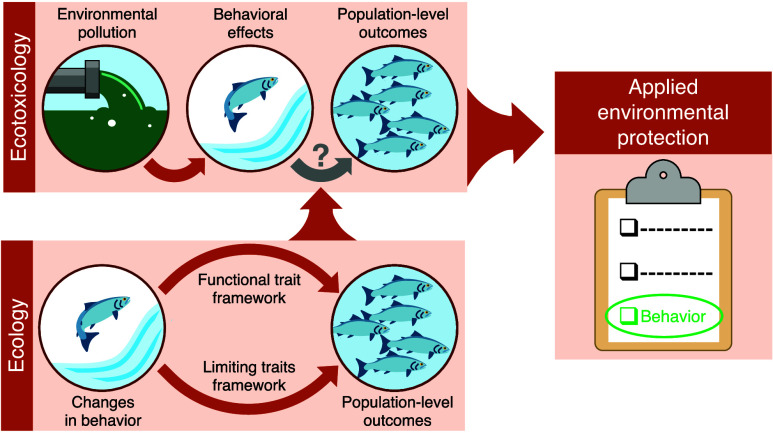

## Introduction

While research investigating the impacts of contaminant exposure on animal behavior dates back to the 1960s,^1^ the field of behavioral ecotoxicology has seen particularly rapid growth over the last two decades (reviewed in refs 2–18 ). Demonstrating this, a recent study synthesizing research on the impacts of pharmaceuticals on aquatic animal behavior identified 901 published studies in that subdiscipline alone, reporting a 19-fold increase in the number of studies published per year between 2007 and 2022.^19^ This surge in research attention is in large part due to methodological and technological advances in recent years that have enhanced the accessibility and reliability of behavioral ecotoxicology research.^15^ In addition, there has been a recent emphasis in behavioral ecotoxicology on improving experimental rigor and transparency, including through the EthoCRED framework reporting recommendations, which aim to reduce the potential for underreporting and information gaps in published studies.^18^

Despite recent progress in the field of behavioral ecotoxicology, behavioral endpoints are still seldom used in applied environmental protection.^20^ Indeed, a recent study identified just six instances where behavioral studies were used, or were at least considered for use, in European Union regulatory decision-making.^21^ This lack of uptake in applied environmental protection persists despite evidence demonstrating that animal behavior can be exceptionally sensitive to disruption by pollutant exposure when compared to conventional endpoints (e.g., mortality, development, reproduction).^9^ Behavior represents the connection between an organism and its environment, meaning that disruptions in the ability to appropriately perform and regulate behaviors can have severe impacts on species ecology and evolution.^22–25^ So, given that the quality and quantity of behavioral ecotoxicology research has increased substantially in recent years, and that behavior is key to the ecology and evolution of species and populations, why do behavioral endpoints remain largely absent from risk assessment and regulatory decision-making?

We use the term “risk assessment” to denote regulatory environmental risk assessment (ERA): prospective (premarket) and retrospective (postmarket) processes used by regulators to combine hazard and exposure. In these assessments, thresholds (e.g., predicted no-effect concentrations, PNECs) are derived from dose–response data—typically using classical endpoints such as mortality, growth, and reproduction, and are then compared with exposure estimates (predicted environmental concentrations, PECs) to characterize risk deterministically, often via risk quotients (PEC/PNEC). Examples of chemical regulations under which ERAs are performed include the EU REACH regulation and the Plant Protection Products Regulation, as well as the U.S. Toxic Substances Control Act (TSCA). The regulations and associated guidance documents set ERA rules and translate ERA outcomes into decisions (e.g., authorization, restrictions, monitoring). Factors that have hindered the incorporation of behavioral data into environmental risk assessment and chemical regulation include a lack of standardization in academic studies (see ref 18), limited familiarity among risk assessors and regulators with regard to behavioral endpoints (see ref 20), and technical and analytical challenges (see ref 15). We also recognize that current ERA includes threshold-based dose–response models and probabilistic Species Sensitivity Distributions (SSDs) that are often supported by classical apical endpoints such as survival, growth, and reproduction compiled in databases (e.g., the ECOTOXicology Knowledgebase, ECOTOX^26^), whereas many behavioral studies still test few concentrations (see ref 19) and some chemicals show nonlinear responses (see ref 27)—issues that complicate, but need not preclude, regulatory integration. However, perhaps the most critical unresolved issue is the uncertainty among some stakeholders about whether and how individual-level behavioral changes are linked to population-level outcomes. Underscoring this, a recent survey of academic, government, and industry scientists on the perceptions and role of behavioral (eco)­toxicology in environmental protection found that 35% of respondents disagreed or strongly disagreed and 30% were neutral with regard to the statement “Behavioral endpoints are easily linked to apical endpoints (growth, reproduction, mortality) and population-level effects.”^28^ This link to population-level outcomes is vital given that environmental assessments almost always consider population-level effects, unlike human health assessments that typically prioritize individual-level protection.^21^

To address this uncertainty, we first review the state of the evidence within behavioral ecotoxicology concerning a link between individual- and population-level impacts, including highlighting recent field-based studies directly demonstrating the impacts of pollutant exposure on fitness-related behaviors and population-level outcomes in the wild. We then consider evidence from neighboring fields, including behavioral ecology, evolutionary biology, population biology, and community ecology, which is rarely considered in this context but stands to provide valuable insights. Finally, we discuss whether the disruption of certain behavioral endpoints may be especially likely to result in population-level impacts, drawing on evidence both from behavioral ecotoxicology and neighboring fields.

### State of the Evidence within Behavioral Ecotoxicology

Over the last 25 years, more than 3600 publications have explored whether and how environmental contaminants—including pesticides, pharmaceuticals, and metals—can alter animal behavior.^18^ These studies span a diverse range of taxa and reveal a wide array of behavioral impacts, including altered patterns of movement, risk-taking behavior, aggression, sociality, reproduction, foraging, and antipredator responses (reviewed in refs 15,18 ). Collectively, this body of evidence demonstrates that environmental pollutants have the potential to interfere with virtually all dimensions of animal behavior.

Laboratory studies, in particular, have been instrumental in identifying pollutant-induced effects on animal behavior. For instance, neurotoxic insecticides such as neonicotinoids can impair bee locomotion, foraging, and navigation, thereby diminishing critical pollination services,^29–32^ while metals like copper can disrupt fish olfaction, reducing detection of food and predator cues and increasing predation risks.^33–36^ Further, endocrine-disrupting chemicals found in wastewater effluents have been shown to suppress essential reproductive behaviors in fish, such as courtship and nest-building, thus compromising reproductive success.^8,37^ Similarly, fish exposed to trace levels of psychoactive pharmaceuticals—such as antidepressants and antianxiety medications—exhibit altered antipredator behavior, increased risk-taking, and reduced social cohesion.^38–40^ The erosion of natural antipredator behavior resulting from exposure to such pollutants has been associated with increased predation risk in a range of species.^41–46^ One striking example projected a 60% decline in the abundance of adult fathead minnows (*Pimephales promelas*) in a population exposed to the endocrine disruptor 17β-oestradiol (E2), based on increased larval predation due to behavioral impairment.^44^ Notably, many of these effects occur at pollutant concentrations that are well below lethal thresholds, underscoring the value of behavior as a sensitive indicator of environmental stress.^7,9^

There has been a significant push in recent years for researchers to employ environmentally realistic exposures and behavioral observations in the field to validate laboratory-based findings, thereby generating a more direct link to real-world outcomes.^15,47^ For instance, a recent study tracking juvenile Atlantic salmon (*Salmo salar*) during river-to-sea migration revealed that benzodiazepine pharmaceutical exposure altered migratory success, in addition to impacting anxiety-like behaviors and sociality in the laboratory.^48^ Indeed, a growing number of studies have demonstrated pharmaceutical-induced disruptions in fish movement and behavior in natural settings, including ponds,^49^ lakes,^50^ and rivers.^51^ Further, field-based research in terrestrial systems has revealed that white-crowned sparrows (*Zonotrichia leucophrys*) exposed to neonicotinoids exhibited rapid declines in food intake and impaired migratory performance, with likely subsequent effects on fitness and survival.^52^ Similarly, sublethal lead exposure in wild golden eagles (*Aquila chrysaetos*) reduced flight height and movement rates and was associated with elevated mortality risk.^53^ Although field-based studies in this research area are still relatively uncommon, the available evidence demonstrates that exposure to environmentally realistic contaminant concentrations can alter animal behavior in the wild, with implied as well as demonstrated consequences for fitness.

Despite this, unequivocally linking pollution-induced behavioral changes to population-level outcomes—such as declines in population size, altered growth trajectories, or demographic shifts within the complexity of real-world environments—remains one of the most pressing and complicated challenges in behavioral ecotoxicology.^14,15^ From a logistical perspective, establishing such connections often requires long-term, large-scale monitoring of individual animals, populations, and communities, which is both resource-intensive and difficult to experimentally control. Isolating the specific contribution of pollutant-induced behavioral disruptions from disturbances produced by other human-induced stressors (e.g., noise, heat, and light pollution, habitat loss, climate change) also complicates (causal) inference. This challenge is amplified because contaminants can alter behavior in multiple interacting species simultaneously,^45,54^ making net system responses more challenging to predict (discussed in refs 14,55 ). These constraints mean that, while behavioral endpoints are highly sensitive and ecologically meaningful, translating individual-level disruptions into clear predictions of population-level impacts requires innovative study designs, integrative population modeling approaches, and strong interdisciplinary collaboration. In this regard, several recent methodological and technological advances offer promising pathways forward.^15,55^ These include (1) the integration of advancements in automated tracking and remote sensing technologies for large-scale behavioral monitoring in natural environments,^56^ (2) the further application of Adverse Outcome Pathways (AOPs) to mechanistically link molecular initiating events with behavioral and fitness-related outcomes,^57^ and (3) the use of individual-based and agent-based models to predict population dynamics based on behavioral metrics.^58,59^ Embracing these innovations is expected to further strengthen the link between individual-level behavioral changes and population-level disruptions.

### Evidence from Neighboring Fields in the Life Sciences

Research in the life sciences over the last three decades has established the importance of animal behavior in influencing individual fitness, population dynamics, and even ecosystem-level processes.^60–63^

Numerous studies in behavioral and evolutionary ecology have demonstrated that behavioral traits are directly linked to individual survival and reproductive success. For example, bolder roach (*Rutilus rutilus*) experienced increased survival in the presence of predatory northern pike (*Esox lucius*) compared to their shyer conspecifics.^64^ Such findings have been reinforced by several meta-analyses linking individual risk-taking behavior (e.g., active, explorative, aggressive, and bold behaviors) to survival in the wild.^65–67^ Similarly, behavioral changes have been shown to influence disease dynamics and animal health.^68–70^ Studies in wood frogs (*Lithobates sylvaticus*), for instance, report that individual activity influenced parasite load^71^ and infection susceptibility,^72^ with research across 227 wetlands indicating that the individual behavioral traits of amphibian hosts can influence infection success and parasite aggregation.^68^ Animal behavior can also mediate mating dynamics and reproductive performance. Indeed, behavior is often central to reproductive success through its involvement in courtship, mate choice, and parental care,^73–78^ with a meta-analysis demonstrating that individuals with more risk-prone behavioral strategies experience higher reproductive success than their risk-averse counterparts.^65,67^ Collectively, this research and numerous other studies conducted over the last several decades have established that animal behavior is integral to understanding variation in reproduction and survival.

Importantly, the fitness consequences of individual behaviors can influence population-level outcomes.^60,79^ Behavioral processes, such as movement and habitat selection, fundamentally drive dispersal decisions and are therefore central to population dynamics.^80–83^ Much work has documented behavior-dependent dispersal patterns and habitat selection in a wide range of species, including birds,^84–87^ fish,^88–91^ mammals,^92–94^ reptiles,^95–98^ and invertebrates.^99–101^ Further, modeling studies have demonstrated that such movement effects can lead to eco-evolutionary consequences for local populations and changes to metapopulation structure.^102,103^ For example, a genetic correlation between aggression and dispersal ability in western bluebirds (*Sialia mexicana*) resulted in more recently established populations at the edge of the species range being more aggressive.^85,104^ These highly aggressive populations, in turn, displaced the resident mountain bluebirds (*Sialia currucoides*), leading to reduced competition and increases in the size of local western bluebird populations.^85^ Similar analyses of 44 bird species reported higher risk-taking behavior in birds from human-modified habitats, potentially facilitating population growth and resulting in larger population sizes in urban environments.^105^ Studies have also shown that individual reproductive decisions can influence local population dynamics. For instance, avoidance of sites occupied by predators during egg laying in adult female mosquitoes (*Culiseta longiareolata*) resulted in greater adult population sizes.^106^ Similarly, population modeling of Amur bitterling fish (*Rhodeus sericeus*) demonstrated that behavioral decisions that alter reproductive success can ultimately influence local population sizes^107^—emphasizing how individual behaviors, through their link with variation in individual fitness, can scale up to impact population processes.

The consequences of altered reproductive behaviors on population dynamics are exacerbated in an increasingly changing world. For example, numerous studies have shown that species often exhibit breeding habitat preferences for human-altered environments that can reduce individual fitness (i.e., ecological traps), conceivably with important population-level consequences.^108,109^ Indeed, preferences of Montagu’s harriers (*Circus pygargus*) for nesting within agricultural fields were associated with increased breeding failure and likely explain the almost 80% decline in their population size over the last 20 years.^110^ Similarly, many aquatic ovipositing insects show greater behavioral preferences for laying their eggs on solar panels than in water bodies due to their reflectance properties, ultimately leading to reproductive failure and population declines.^111^ Human-driven shifts in mating behaviors can also substantially alter population composition.^112^ A case in point is seen in changes to chorusing behavior and mating calls in human-modified habitats, which is suspected to have resulted in the hybridization of two closely related songbirds in urban environments.^113^

Altered animal behavior can not only influence population dynamics but can ultimately affect community structure and ecosystem dynamics.^114–116^ For example, less-social western mosquitofish (*Gambusia affinis*) groups reduced initial prey density to a greater extent than more-social groups.^117^ Similarly, more-active predatory dragonfly nymphs (*Epitheca canis*) disproportionately reduced the abundance of zooplankton when compared to less-active conspecifics.^118^ Predators are also known to cause shifts in the behavior and habitat use of prey via nonconsumptive effects, resulting in changes to nutrient cycling and community dynamics.^119,120^ Indeed, broader work has now emphasized the role of animal behavior in influencing trophic interactions,^121^ pollination and seed dispersal,^114,122^ information flow within populations and ecological networks,^123,124^ and the success of biological invasions^125,126^ —further highlighting how behavioral changes at the individual level can scale up to affect population-level outcomes and even community and ecosystem dynamics.

### Are Certain Behaviors *More Relevant* at the Population Level?

Behavior can be a powerful indicator of individual performance and fitness—and, by extension, broader outcomes at the population, community, and ecosystem levels.^7,127^ However, not all behavioral traits are equally predictive of demographic trends. To evaluate which behaviors should be prioritized in ecotoxicological research and regulatory efforts, we propose combining insights from two complementary ecological frameworks: the functional trait framework and the limiting traits framework.

The functional trait framework, widely applied in community and trait-based ecology, provides a system for classifying traits based on their role in survival and reproduction.^128,129^ In this framework, behaviors can be placed along a gradient of fitness relevance. At one end are “functional trait proxies,” such as general locomotor activity and exploration, group cohesion and shoaling tendency, light attraction/avoidance, and startle-response latency, which tend to correlate with fitness via indirect, context-dependent pathways. In the middle are “functional traits” such as foraging efficiency and prey-capture success, courtship behavior and mate choice, and antipredator behavior, which influence key processes (e.g., energy acquisition, mating success, predator avoidance) that affect performance. At the other end of the gradient are “performance traits,” such as survival, daily food-intake rate, growth, migration success, and number of offspring produced, which directly determine fitness. This framework also acknowledges that trait importance is shaped by the ecological context. For instance, activity level may be more important—and more tightly linked with fitness—in high-predator-risk environments than in low-predator-risk environments.^128^ While ecologically informative, the functional trait framework alone does not identify which traits are the most sensitive or consequential under chemical stress.

The limiting traits framework, developed in behavioral ecology, addresses this shortcoming by focusing on traits (termed “limiting traits”) that constrain population performance under stress (termed “limiting factor,” e.g., contaminant exposure).^130^ The framework emphasizes that trait sensitivity and ecological consequences may vary across demographic windows (termed “limiting demographic window”), highlighting the importance of key life-history stages (e.g., migration, first reproduction), where behavioral disruptions can have disproportionately large effects on population dynamics.^131^ This idea aligns with principles from population ecology such as elasticity analysis, which identifies life stages with the greatest influence on population growth.^131^ Importantly, this framework also considers the timing of behavioral disruption relative to population-level consequences (termed “latency of effect”). Immediate impairments, such as a failure to avoid predators, can lead to rapid and observable demographic declines, whereas delayed effects, such as subtle reproductive changes, may be harder to detect. In this regard, it is important to note that a key behavior-mediated route to population-level consequences operates via sexual selection. Pollutants can alter courtship signaling, mate choice, and postcopulatory processes, and individuals may differ in the extent to which their reproductive behavior is impaired under exposure (reviewed in refs 37,112,132 ). Such genotype-by-environment effects can reshape the variance in male and female reproductive success, skewing who breeds and how much, thereby potentially narrowing the genetic mix carried forward and eroding genetic diversity over time. However, despite their clear importance, such delayed consequences may be weighted less heavily than immediate ones, a concept referred to as “temporal discounting” borrowed from behavioral ecology and decision theory.^133,134^ From both ecological and regulatory perspectives, behavioral traits with immediate and measurable fitness impacts may therefore be the most predictive and actionable. In addition, the limiting traits approach asks why such fitness-limiting behaviors persist. Constraints such as developmental plasticity or phylogenetic conservatism may explain why populations cannot readily adapt to certain stressors, even when traits are closely linked to fitness.^135^ As such, the framework integrates mechanistic, ecological, and evolutionary insights.

Together, these two frameworks offer a two-tiered strategy for identifying behavioral endpoints of regulatory relevance (Figure 1). The functional trait framework provides an ecological classification system, helping to position behaviors along a gradient of fitness relevance, from proxies to direct fitness components. It tells us which behaviors matter in ecological terms, and in which context. The limiting traits framework contributes a stress-oriented focus on vulnerability, aiding the identification of behaviors that are the most likely to mediate population-level responses under chemical stress. It is particularly concerned with identifying traits that are the most sensitive, least compensable, or most influential during vulnerable life-history stages. Combining these two frameworks may help researchers, risk assessors, and regulators to move beyond endpoint inventories and toward a more ecologically meaningful and evidence-based prioritization of behavioral endpoints that are relevant for environmental protection.

**Figure 1:**
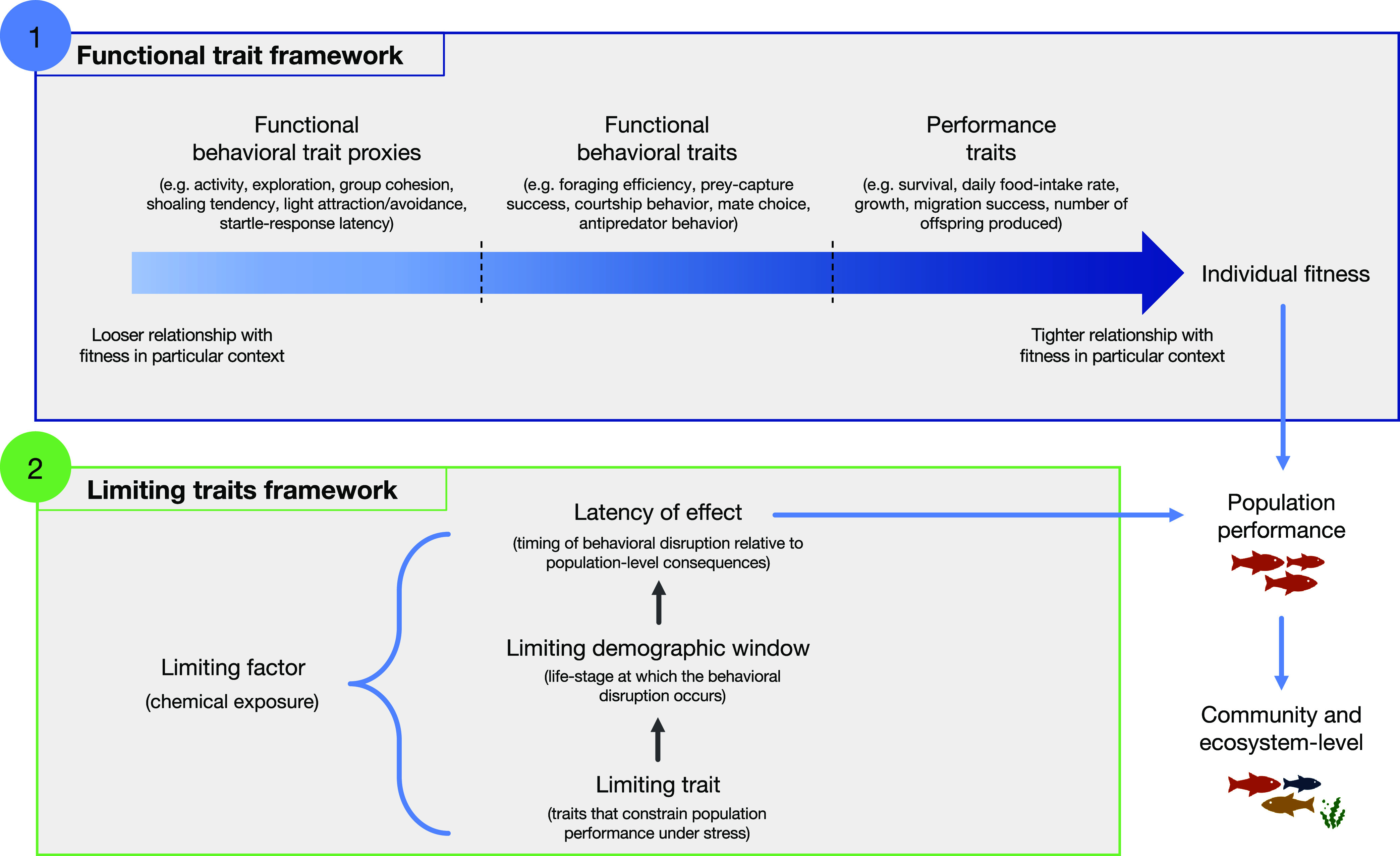
A two-tiered strategy combining the functional trait (1) and limiting traits (2) frameworks to identify those behavioral traits during key life-history stages that are the most tightly linked with fitness (functional trait framework) and whose disruption is the most immediate and consequential at the population level (limiting traits framework), typically with flow-on effects at the community and ecosystem levels. Such traits can be considered to be the most predictive and actionable. From a risk assessment and regulatory perspective, combining these frameworks can be used to predict the likely population-level impacts of contaminant-induced behavioral changes. As two hypothetical examples: (i) a study reporting light avoidance (a functional behavioral trait proxy) in postspawning adult fish (a relatively nonlimiting demographic window) which occurs after one generation (high latency of effect) of chemical exposure would be considered to have a relatively low probability of affecting population-level outcomes; (ii) a study finding reduced daily food-intake rate (performance trait) in juvenile birds (limiting demographic window) that occurs immediately after 1 week (low latency of effect) of exposure to a contaminant would be considered to have a relatively high probability of having population-level impacts.

Host–parasite systems provide an illustration of how behaviors expressed in a single context can span the functional-trait gradient while simultaneously acting as limiting traits. Specifically, parasites such as trematodes and acanthocephalans manipulate host behavior to enhance their transmission.^136–139^ In crustacean hosts, these parasites modulate serotonin pathways to alter phototaxis—causing infected individuals to swim toward light (a functional trait proxy), thereby increasing their visibility and decreasing their avoidance of fish predators (a functional trait once it is shown to elevate predation risk^140^). Predation rates on infected individuals can be 10- to 28-fold higher than on uninfected individuals, with the resulting survival differential comprising a performance trait.^141,142^ Moreover, this phototactic switch is expressed only during the parasite’s transmission window—the point at which the parasite’s survival depends on its host being consumed by the correct predator. Because this behavior is highly susceptible to disruption during that crucial stage, it fulfills the limiting-traits criterion of a behavior that is both especially sensitive to disturbance and expressed at a stage when even small changes can have outsized population-level effects. These studies not only demonstrate the fitness consequences of altered behavior but have directly inspired research into how serotonin-modulating pharmaceuticals, including various antidepressants, can similarly influence invertebrate behavior and predation risk.^143–145^

### Implications

When considering the evidence both within behavioral ecotoxicology and across related disciplines in the life sciences, it is clear that disruption of animal behavior due to pollutant exposure can be expected to result in population-level consequences and even have far-reaching impacts on communities and ecosystems. By aligning behavioral ecotoxicity testing and findings with established ecological theory, such as the functional trait and limiting traits frameworks, researchers and regulators can better prioritize those behavioral endpoints with the highest predictive relevance. Moreover, integrating behavioral approaches into both risk assessments and regulatory testing will strengthen environmental protection by ensuring that sublethal yet consequential effects are not overlooked.
